# Reducing Wait Time in a High-volume Pediatric Neuro-oncology Clinic by Optimizing Process Flow: A Quality Improvement Project

**DOI:** 10.1097/pq9.0000000000000557

**Published:** 2022-06-14

**Authors:** Anna Vinitsky, Barbara David, Layna Michalik, Nicole Ramirez, Adam Risinger, Jonathan D. Burlison, Jacky Zanders, Bridget Mans, Katie Heady, Joni Holdiness, Ibrahim Qaddoumi, Giles W. Robinson, Daniel Moreira, Santhosh A. Upadhyaya, Amar Gajjar

**Affiliations:** From the * Department of Oncology, St. Jude Children’s Research Hospital, Memphis, TN; † Surgical Services, St. Jude Children’s Research Hospital, Memphis, TN; ‡ Center for Advanced Practice, St. Jude Children’s Research Hospital, Memphis, TN; § Ambulatory Operations, St. Jude Children’s Research Hospital, Memphis, TN; ¶ Department of Pharmaceutical Science, St. Jude Children’s Research Hospital, Memphis, TN; ∥ Inpatient Units, St. Jude Children’s Research Hospital, Memphis, TN; ** Outpatient Clinics, St. Jude Children’s Research Hospital, Memphis, TN; †† Nursing Administration, St. Jude Children’s Research Hospital, Memphis, TN; ‡‡ Global Pediatric Medicine, St. Jude Children’s Research Hospital, Memphis, TN.

## Abstract

**Introduction:**

Hospital wait time (WT) influences healthcare quality and patient satisfaction. Long WTs are distressful for patients and considered substandard healthcare delivery. Pediatric hematology/oncology patients with complex medical conditions frequently need multiple appointments in a day, making their scheduling very challenging. Here, we report a quality improvement (QI) project aimed to decrease the percentage of patients waiting >30 minutes before room placement in the neuro-oncology clinic.

**Methods:**

We measured WT from when the patient reported to the clinic (or, for those arriving early, from scheduled appointment) to when the patient got an exam room. We collected data by random sampling and collected baseline data over the initial 4 weeks; generated process mapping and Pareto charts to identify reasons for delayed patient placement in rooms; and used iterative Plan-Do-Study-Act (PDSA) cycles to test interventions. We used Run charts and Shewhart charts for data analysis.

**Results:**

Our baseline data analyses showed provider and room availability as critical reasons for delayed room placement (38.4% and 30%, respectively). We also completed related PDSA improvement cycles. The median percent of patients waiting >30 minutes decreased from 21% to 13%. The median average waiting time decreased from 21 to 11 minutes.

**Conclusion:**

Using structured QI methodology, we decreased the percent of patients waiting >30 minutes before room placement and overall WTs. We developed a strategy for continuous improvement and future interventions. Furthermore, our results suggest that QI projects, which account for the complexity of hospital systems, can improve patient flow throughout the hospital.

## INTRODUCTION

Efficiency is among the 6 domains of healthcare quality, and patient wait time (WT) can be a source of inefficiency in healthcare systems.^1,2^ Consequences of longer WTs include lack of confidence in the provider, negative perception of the quality of care received, and possible negative outcomes.^3,4^ Pediatric Hematology-Oncology patients with complex medical conditions frequently need multiple daily appointments, which presents scheduling challenges. Recent studies show that formal improvement methods can help define and implement practical solutions for patient WTs.^5,6^

In our busy outpatient multidisciplinary pediatric neuro-oncology (NO) clinic, patients routinely spend more than 30 minutes waiting before being seen by a provider. Our project aims to decrease the percentage of patients waiting >30 minutes before getting room placement in the NO clinic from 21% to 5% within 1 year of initiating the project. This initiative was approved by the St. Jude Children’s Research Hospital (St. Jude) Institutional Review Board as a quality improvement (QI) project. The Standards for Quality Reporting Excellence guidelines for QI reporting were used to report results.^7^

## METHODS

### Context

St. Jude is a 77-bed pediatric hospital with integrated outpatient clinics offering subspecialty and surgical services for children with cancer, blood disorders, and other serious diseases. It has ~3,500 inpatient admissions and sees ~180,000 outpatient visits annually. An average of 7,500 visits are scheduled at the NO clinic per year, and it has 6 clinic rooms and 1 consult room. There is a centralized assessment and triage (A&T) area where all patients have vital signs checked and blood drawn before their appointments. In addition, there is a dedicated space (“medicine room”) to administer outpatient chemotherapy and transfusion of blood products. All subspecialty clinics use the A&T area and medicine room. In addition to primary appointments with the neuro-oncology team, we schedule multiple ancillary appointments in NO clinic rooms for patients when needed (eg, nutritionist, Pharm D, research nurse). Patients in isolation (contact or droplet) are required to stay in the NO clinic room for all scheduled appointments, including but not limited to appointments with a rehabilitation specialist, psychologist, or school teacher. These appointments are normally scheduled in a respective subspecialty clinic. We also see patients with previously scheduled appointments and acute issues in the NO clinic as add-on visits.

The NO clinic staff includes 6 neuro-oncologists, 5 advanced practice providers (APPs), 5 registered nurses (RNs), 1 certified nurse assistant, and 1–2 front office staff members. Each patient is assigned to a primary team that includes a pediatric neuro-oncologist, APP, and RN. Each team has assigned clinic days (usually 2 days/week). Two to 5 teams are scheduled to see patients in the NO clinic on any day. We preferentially schedule all patients with complex medical issues with their primary team for continuity of care. Several teams have higher patient loads; therefore, we schedule more patients to be seen on certain days of the week. APPs or fellows see most patients at the beginning of the visit, and an attending physician evaluates patients at the end. Some consult visits (eg, discussing informed consent) are performed by attending physicians. This workflow requires maintaining multiple provider templates used for patient scheduling. We have no restrictive directions for the scheduling team. Therefore, overbooking is frequent for primary teams with the highest patient load. Furthermore, patients developing acute issues and requiring same-day evaluation are assigned as “add-on” visits and seen by the primary team (when available) or by the “doctor of the day.” Two days per week (2–3 h/d), NO clinic rooms are provided to the neurosurgery team to see mutual patients.

### Interventions

**Fig. 1.**
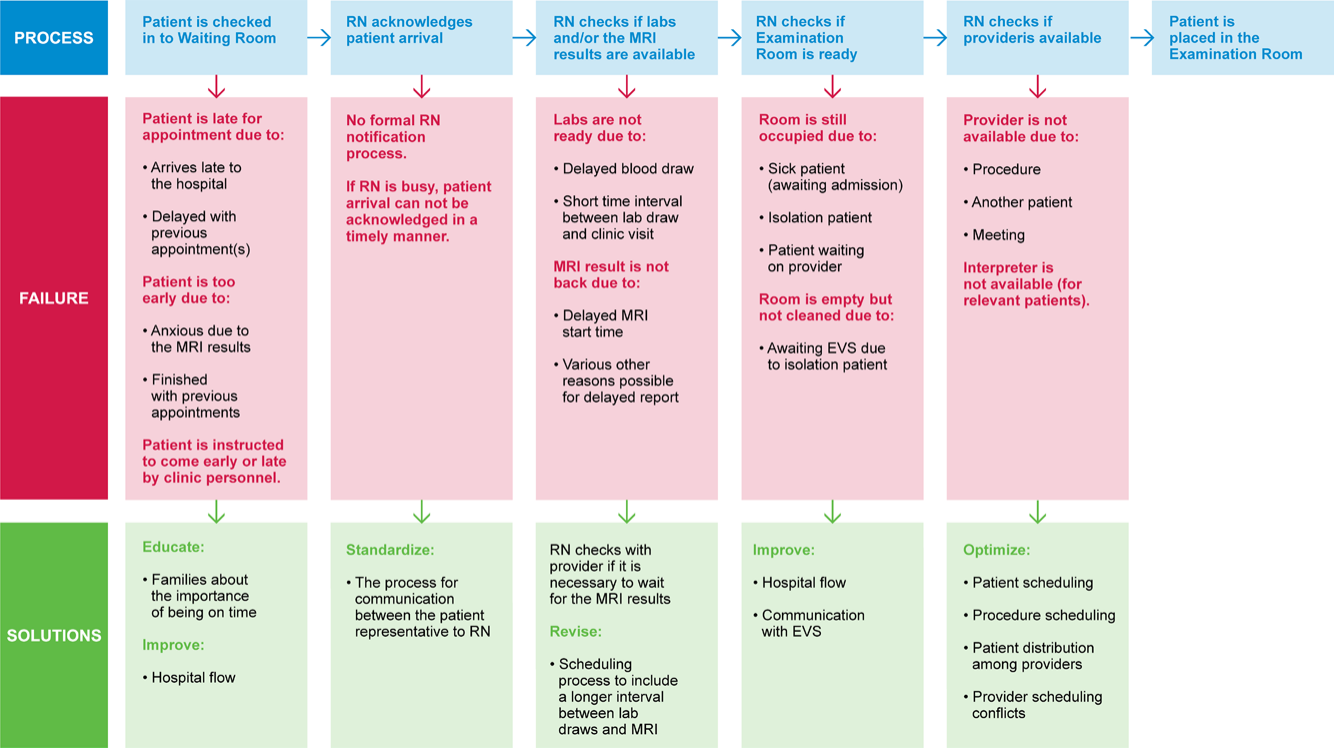
Process flow for patient arrival to the examination room in the NO clinic. EVS, environmental services; Labs, laboratory results; MRI, magnetic resonance imaging; RN, registered nurse.

**Fig. 2.**
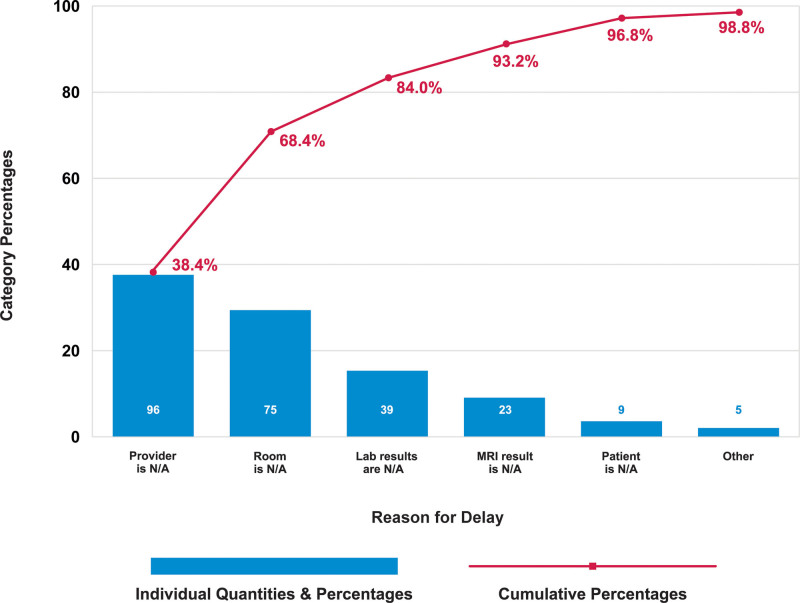
Pareto diagram showing reasons for delayed room placement in the NO clinic. Lab, laboratory; MRI, magnetic resonance imaging; N/A, not available; NO, neuro-oncology.

**Fig. 3.**
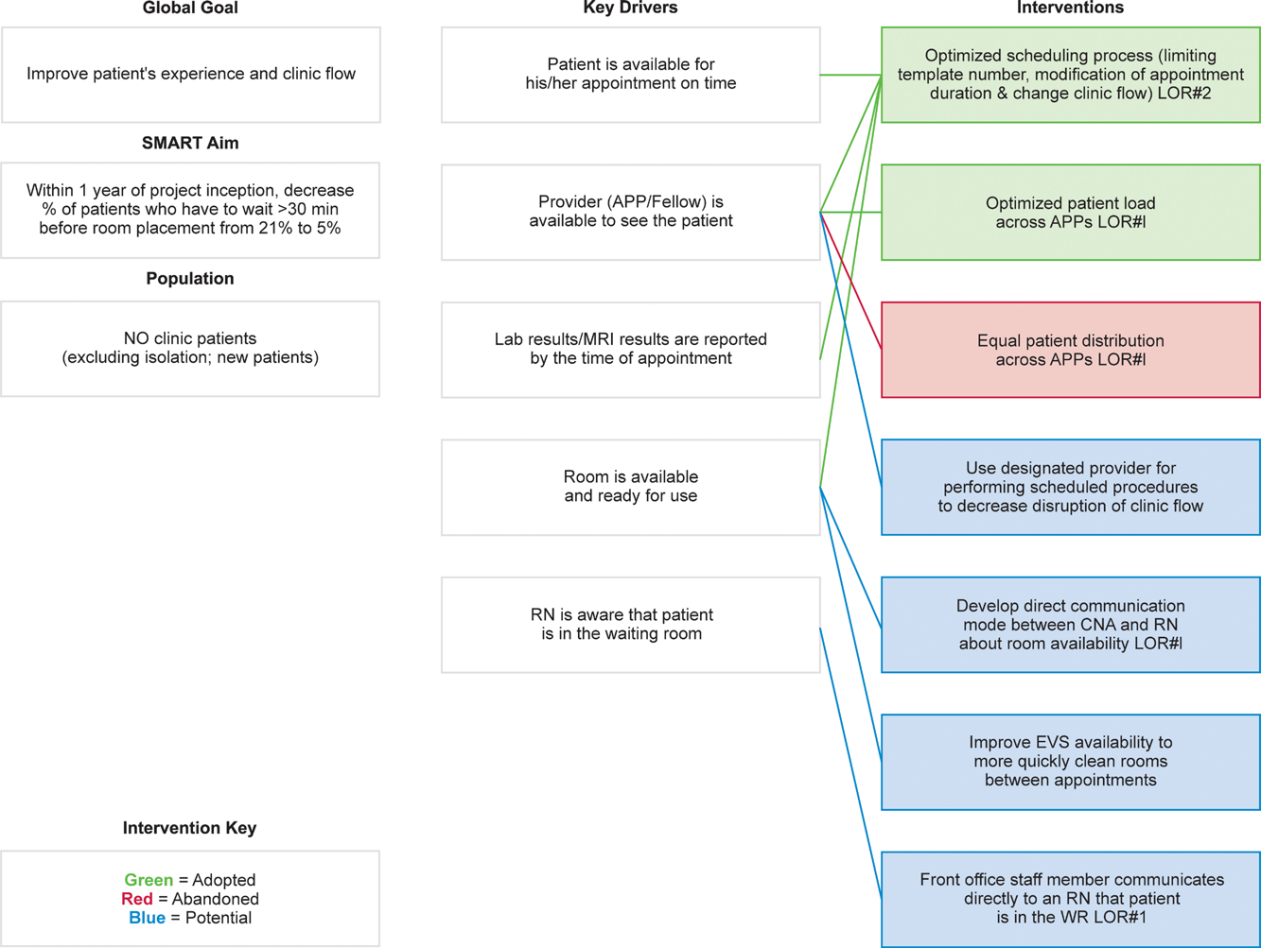
Neuro-oncology wait time key driver diagram demonstrates the goals/aims, drivers to achieve goals, and adopted, abandoned, and potential interventions. APP, advanced practice provider; CAN, certified nursing assistant; LOR, level of reliability; MRI, magnetic resonance imaging; NO, neuro-oncology; RN, registered nurse; WR-waiting room.

We assembled a multidisciplinary team of physicians, APPs, nurses, schedulers, and representatives from clinical operations. We generated a process flow map that outlined the patient’s journey from “check-in” at registration to placement into a NO clinic exam room. Figure 1 summarizes process steps, possible failures, and potential interventions. We also used a Pareto chart to identify the most common reasons for the late arrival of patients to the clinic and delayed room placement (Fig. 2). Our team identified key drivers and potential interventions needed for timely room placement and summarized them in the Key Driver Diagram (KDD, Fig. 3). We used Iterative Plan-Do-Study-Act (PDSA) cycles to test interventions.

### Baseline Evaluation

For a baseline, we collected data for each clinic visit over the first 4 weeks of the project (n = 603). We excluded patients in isolation (n = 30), new patients in the clinic (n = 6), and those not showing up for their appointment (n = 10). Of 556 patients, only 127 (23%) came for their visit “on time” (defined as arrival within 10 minutes before an appointment time). Two hundred sixty-three patients (263, 47%) checked into the waiting room before their scheduled appointment, and 166 (30%) were late. We identified that the most common reason for delayed arrival to the NO clinic was previous appointments in other hospital areas (63%) and late arrival to the hospital (23%). Late appointment arrivals were mainly generated from delays in A&T (35%) and MRI areas (18%).

Figure 2 summarizes the reasons for delayed room placement in the NO clinic: provider availability and room availability were the most common reasons for delayed room placements (cumulative 68%). We identified that provider unavailability was mainly due to them being occupied with another patient or doing a procedure. Baseline data helped identify key drivers and interventions focused on provider and room availability (Fig. 3).

### Targeted Interventions

#### Increased “Provider Availability”

The unavailability of an assigned provider at the visit was the main reason for patients’ room placement delay (Fig. 2). In most cases, the assigned provider was busy seeing another patient (38.5%) or charting (31%). At baseline, all patients were assigned to their primary APP/fellow without considering the anticipated patient load for the day. Hence, providers were expected to see more patients on their “primary clinic” days and have a significantly lower patient load on their “nonprimary clinic day.” Frequently ≥2 patients were scheduled simultaneously for the same provider, as we have no restrictions on the total number of patients scheduled for the day.

We tested 2 interventions: (1) “Optimized patient load across APPs,” when the number of patients assigned to a “primary” provider was based on realistic expectations (eg, all overbooked patients were reassigned to another available provider who did not have their primary clinic day). However, in this scenario, the primary provider still had a higher patient load during his/her clinic days. (2) “Equal patient distribution across APPs” when all scheduled patients for the day were distributed evenly among all available providers without strict adherence to primary team assignment. In addition, we made several modifications to our scheduling templates (see next section) to improve provider availability.

#### Increased “Room Availability”

The optimizing scheduling process included several simultaneous interventions that focused on improving “room availability.”

1. Limiting provider templates: At baseline, our schedulers used many template types. Each patient was scheduled for a “provider” and booked to a random “clinic room” template. There were 17 scheduling templates available (6 templates for each attending MD, 5 templates for APPs, and 6 for clinic rooms). This number of templates created a false impression of having ample “booking space.” To resolve this issue, our team developed a new scheduling template limited to the number of available exam rooms. Each primary team was assigned 1 or 2 rooms (depending on the expected patient load for the day), and all their patients were scheduled in available open slots within this room block. This new template provided an overview of available appointments for each room/provider and helped avoid overbooking and distributing patients more evenly throughout the day.
2. Modifying appointment duration: Appointment durations were modified to reflect the time required for a visit based on the appointment type. For example, the time allocated for a “new patient” visit was increased from 45 minutes to 120 minutes; the duration of the “on therapy” and “off therapy” appointments was decreased from 45 minutes to 30 minutes, and the “lab check” visits were changed to 15 minutes.
3. Change in clinic flow: We instructed primary teams to use dedicated room blocks assigned to their respective teams. Our standard operation model is to share all clinic rooms among teams and use them on a first-come, first-serve basis. Thus, nurses were responsible for checking how many patients were in the waiting room and assigning them to clinic rooms, considering the appointment time. Given that many patients do not report to the clinic at their scheduled time, this approach was considered beneficial in ensuring the flow of patients. However, this left no opportunity to predict how many rooms would be available at a particular time of the day and the best time to schedule an additional patient for a sick visit if needed.

##### Study of Interventions

We sequentially implemented interventions and used the statistical process control chart to assess their impact. We used established rules for differentiating between special and common causes of variation for charts.^8^

### Measures and Data Collection

The primary outcome was the percentage of patients waiting more than 30 minutes before placement in an exam room. We collected WT data (in minutes) and calculated the average WT. Our patients are not placed in a clinic room unless an assigned provider can see the patient. Thus, WT before room placement is equivalent to WT before being seen by a provider.

We defined WT as follows:

- For the patient who arrived for his/her appointment “on time” or “late,” time was calculated from when the patient reported to the clinic’s waiting room to the time that the patient was placed in an exam room.
- For the patient who arrived for the appointment “early,” time was calculated from the patient’s scheduled appointment time to when the patient was placed in an exam room.

We used random sampling techniques to minimize the data collection burden throughout the project. Given the uneven patient load during the day and among weekdays, we sampled patients during morning and afternoon clinic sessions and collected data on each clinic day throughout the week (Monday–Friday). A list with the total number of visits for each day was generated in the morning, and designated staff members picked 2–3 at random from morning and afternoon sessions.

We created a data collection form to capture the time of patient arrival to the waiting room; reason for the delay (if a patient arrived late); time of scheduled appointment; the time when the patient is placed in an exam room; the time when nurse, APP/Fellow, and attending physician enter and exit the room; and time when the patient leaves the exam room. We also recorded the reason for the delay when applicable and available. All NO clinic staff were trained to ensure proper time recording on the timing sheet before data collection.

In addition, the scheduling team monitored if an appointment could not be scheduled at the requested date/time due to the unavailability of “booking space” (balancing measure).

### Analysis

Using statistical process control charts, we analyzed the primary outcome “% of patients waiting more than 30 minutes.” We used a run chart for analysis of WT.^9^ We shifted the centerline based on established rules for special cause variations in the context of interventions.^8^

## RESULTS

**Fig. 4.**
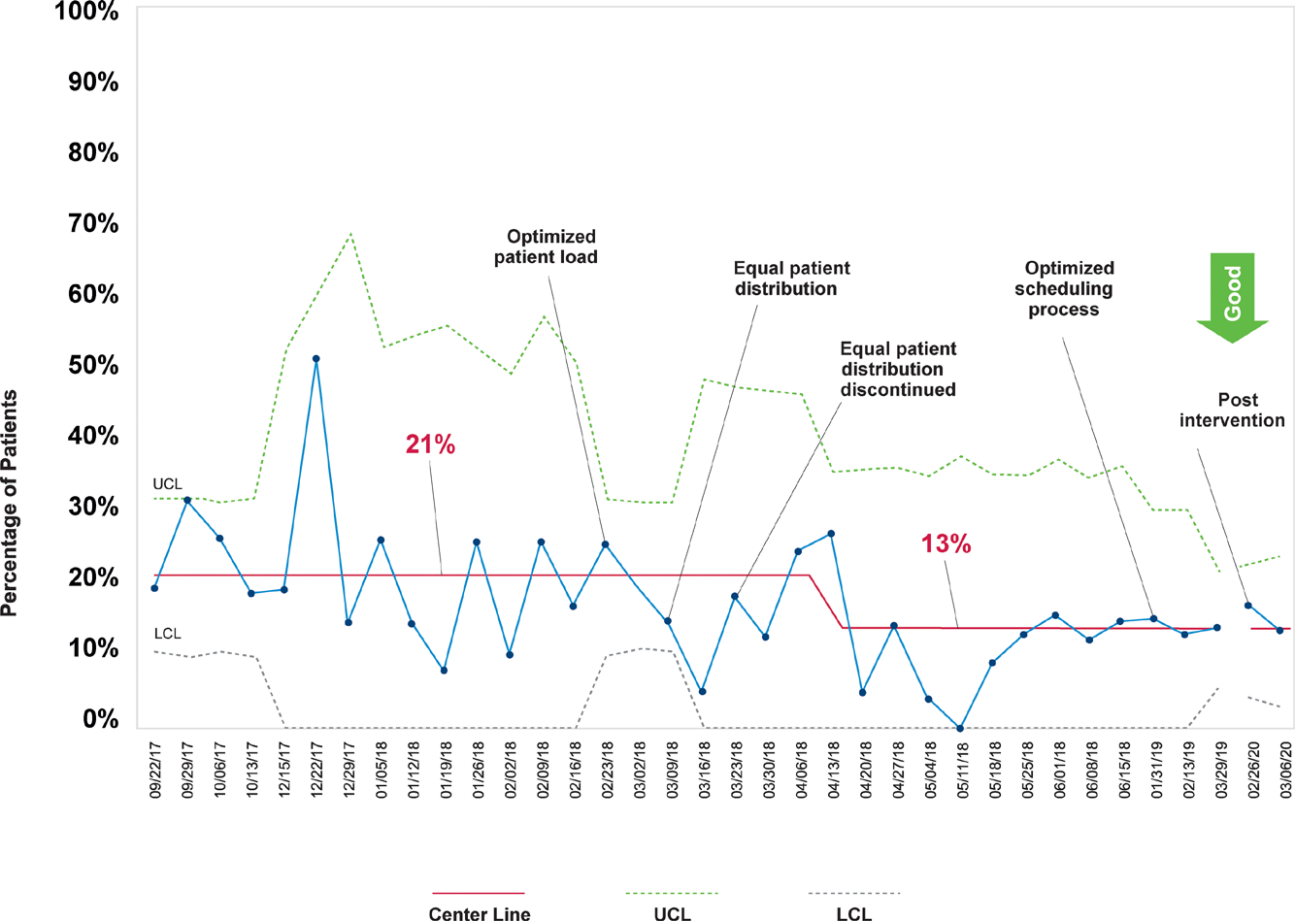
Control chart shows the percentage of patients who waited >30 minutes before room placement.

**Fig. 5.**
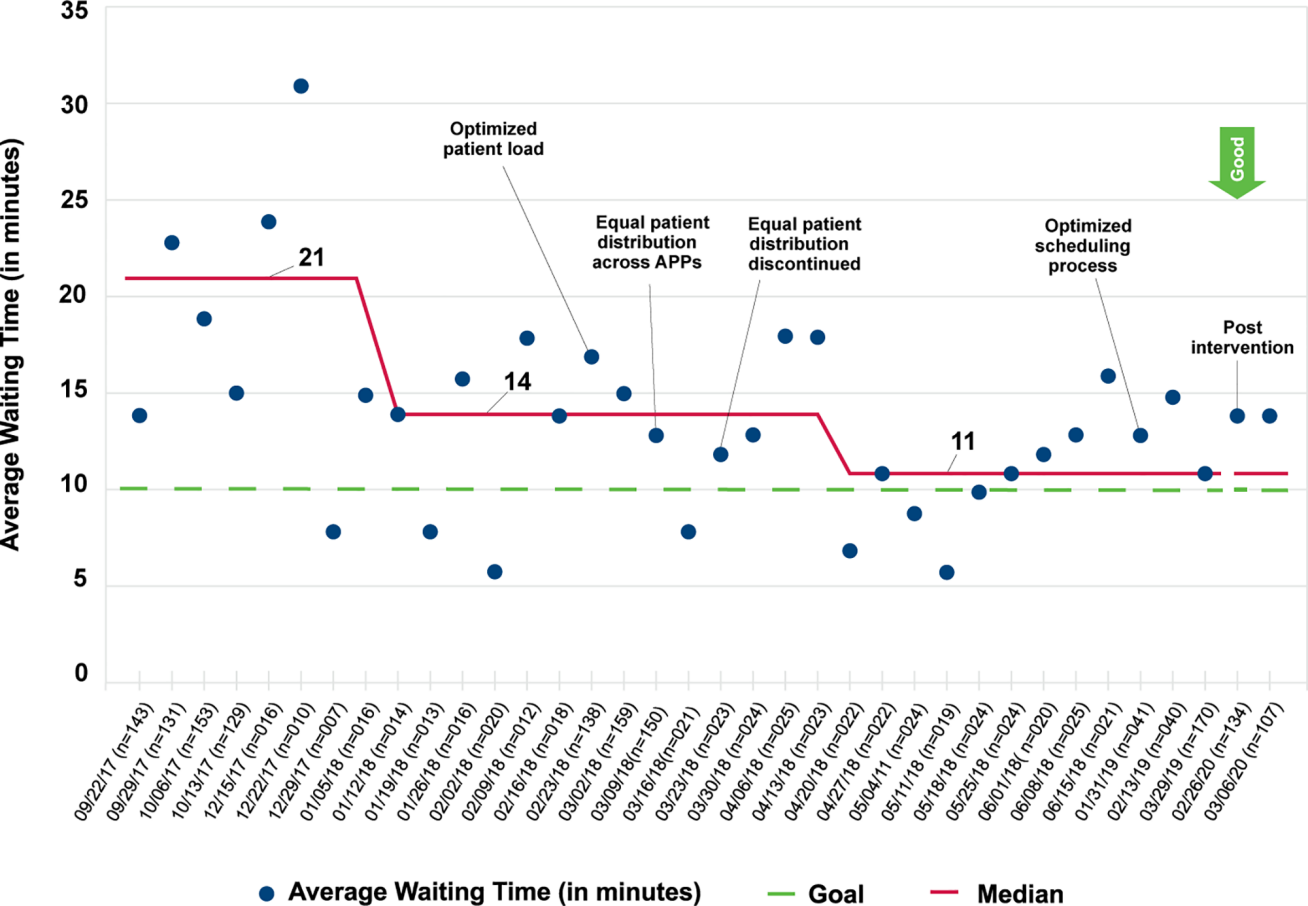
Run chart showing median average waiting time for the patient until room placement in the NO clinic. NO, neuro-oncology.

During the project period (September 2017–March 2020), we observed significant, sustained improvements in the percentage of patients who waited more than 30 minutes, 21% to 13% (Fig. 4). We also documented special cause and a reduction in the median average WT from 21 to 11 minutes (Fig. 5). Both measures also experienced reductions in variability across the project period. Our balancing measure, unavailability of “booking space”, experienced no changes.

We noted a slight decrease in the percentage of patients who waited more than 30 minutes after an initial intervention focused on optimizing patient load among providers (Fig. 4). In addition, we observed a trend toward additional improvements when an equal number of patients were assigned to each APP (Figs. 4, 5).

Our subsequent intervention, optimizing the scheduling process, was tested for 1 week. During this week, the number of patients who waited more than 30 minutes was below 10% for each clinic day except Thursday (considered our busiest clinic day), and the median percent for the week remained at 13%. Based on this result, we made permanent changes in our scheduling clinic templates in June 2019.

Eleven months after the main project, we collected additional data to track sustainment and observed no increases in the percentage of patients waiting >30 minutes (Fig. 4). Although we did not document special cause for median average WT, we noticed it increased slightly (Fig. 5). Unfortunately, the duration of these postproject observations was brief and was discontinued prematurely due to our hospital’s response to COVID-19.

## DISCUSSION

Our project demonstrated that a multidisciplinary team using a structured QI model could accomplish significant improvements in WTs in a busy Pediatric Hematology-Oncology hospital. We used the Model for Improvement framework, which uses a step-by-step approach.^10^

### Lessons Learned

#### Importance of Clear Communication

We realized the importance of clear communication with the clinic team after our first PDSA attempt (focused on assigning primary APPs more patients) did not show the expected improvement. We determined a contributing factor was a lack of detailed instructions on how “optimized patient load” could be obtained. Therefore, we ensured that clear instructions were provided for each subsequent intervention for the remainder of the project.

#### Regular Feedback

Throughout the project, we modified our interventions based on the team’s feedback. For example, we observed the best improvement when we tested “Equal patient distribution across APPs.” However, in further discussions, team members raised concerns that continuity of care may be compromised by assigning a patient to a provider less familiar with the patient. Eventually, we adopted our first intervention, “Optimized patient load across APPs,” wherein a primary APP is assigned a higher patient load on his/her primary clinic days. We realized that providers’ familiarity with details of every primary patient’s treatment course allows them to be more efficient during the visit and is in the best interest of patients. In addition, by optimizing our scheduling process, we minimized other reasons for limited provider availability (eg, frequency of overbooking decreased after changes made in the number of open templates and duration of appointments), which helped ensure the availability of the primary provider for patients.

We also experienced that interventions with a higher reliability level^11^ (ie, embedded into the modified workflow) helped sustain improvement. For example, we made permanent changes in the number of available scheduling templates and appointment duration, which helped ensure that these modifications were used consistently among schedulers. In addition, room availability was the main reason for delayed patient room placement; therefore, we collaborated with hospital operational leadership to create an additional consult space after the project’s completion.

Regarding the change in clinic flow and the use of assigned clinic rooms for primary teams, despite our best intentions, it could not be widely accepted by the clinic staff (mainly RNs) for several reasons. For example, given that a significant number of patients still arrive in the clinic outside their “appointment window” and sometimes appointments run longer (eg, when a physician has to discuss disease progression seen on routine MRI), it is difficult to maintain “planned” room assignment in the middle of a busy clinic day. Despite this, because we could use our optimized scheduling process on the “back end,” we could sustain the improvement.

#### Next Steps

Although we did not achieve our goal, we launched several other hospital-wide initiatives based on data gathered during this project. For example, the APP leadership decided to introduce a “proceduralist” role: having a provider dedicated to performing scheduled procedures to minimize disruption of clinic flow and increase provider availability. Projects on optimizing patient flow in the MRI suite and assessment and triage area are underway. In addition, due to the COVID-19 pandemic, we have implemented Telehealth in the clinic, which allows us to optimize room availability for those patients who need to be seen in the clinic. Our hospital is currently implementing a new Electronic Health Record that will support real-time data collection. Lastly, the ability to automatically collect WTs as one of the performance metrics will allow us to assess the effect of those mentioned above or any new process changes in real-time.

### Limitations

This project has some limitations. St. Jude is a specialized hospital that solely treats children with hematological or oncologic diseases, and our project may not be generalizable to typical pediatric settings. We could not fully implement all our interventions during the project period, which hindered our ability to meet our target goals. One barrier encountered was patients being delayed at other appointments within the hospital, in areas outside the scope and control of our project (eg, centralized assessment and triage area and MRI suite). Several interventions were also not implemented sequentially, making it difficult to discern their impact. Another major barrier encountered was the limited number of clinic rooms. The clinic space was designed many years ago, and since that time, the patient volume has increased, but the number of clinic rooms has remained unchanged. Upon completing the project, we repurposed one of the storage rooms into an additional consult room, which is now used for prolonged consult/result discussion visits. However, due to existing limitations in electronic data collection and limited staffing to perform tedious manual data collection, we could not assess the impact of this addition on waiting time. Also, due to COVID-19 emerging, the clinic’s workflow was vastly altered, and we no longer had the resources to collect project data. Our last collection date was March of 2020.

## CONCLUSIONS

We significantly decreased the percent of patients having a WT of more than 30 minutes before room placement and decreased WTs in our outpatient clinic using structured QI methodology.

We continue to further optimize processes in our clinic using new knowledge gained from this project.

## Disclosure

The authors have no financial interest to declare in relation to the content of this article.

Preliminary data from this study were presented as a poster presentation at the 2019 ASPHO meeting.

## ACKNOWLEDGMENTS

Assistance with the study: The authors thank Vani Shanker, Ph.D., ELS, for editing the article.

This study was supported in part by the American Lebanese Syrian Associated Charities.
